# Complex patellar tendon reconstruction using a peroneus longus autograft and structural cortical bone graft after multiple failures: a case report

**DOI:** 10.1093/jscr/rjag844

**Published:** 2026-09-28

**Authors:** Lucas Rezende, Marcos Paulo Tercziany Vanzin, Luis Antonio de Ridder Bauer, Luis Henrique Longo, Camille Midori Okuyama, Sofia de Almeida Queiroz, Christiano Barbieri de Oliveira Martoni

**Affiliations:** Department of Orthopedics, Universidade Federal do Mato Grosso, Sinop, PR, Brazil; Department of Orthopedics, Hospital do Trabalhador, Curitiba, PR, Brazil; Department of Orthopedics, Hospital do Trabalhador, Curitiba, PR, Brazil; Department of Orthopedics, Hospital do Trabalhador, Curitiba, PR, Brazil; Department of Orthopedics, Hospital do Trabalhador, Curitiba, PR, Brazil; Department of Orthopedics, Hospital do Trabalhador, Curitiba, PR, Brazil; Department of Orthopedics, Hospital do Trabalhador, Curitiba, PR, Brazil

**Keywords:** patellar tendon, rupture, reconstruction, autograft, extensor mechanism

## Abstract

Patellar tendon rupture is an uncommon but disabling injury of the knee extensor mechanism. Chronic rupture and repeated repair failure are associated with tendon degeneration, patella alta, and compromised bone stock, making reconstruction challenging and without a standardized approach. We report a 41-year-old male with spontaneous patellar tendon rupture following tendinopathy, with two failed repairs evolving to re-rupture, patella alta, and severe bone loss of the inferior patellar pole. Reconstruction was performed using suture anchors, structural autologous cortical bone graft from the distal tibia, and augmentation with a peroneus longus tendon autograft. At 26 weeks, the patient achieved full active extension, range of motion from 0° to 120°, improved patellar height, and significant functional recovery. This combined biological and structural approach may represent a viable option in complex revision cases with tendon and bone deficiency.

## Introduction

Patellar tendon rupture compromises the knee extensor mechanism and may result from acute trauma or chronic degeneration [1]. In chronic cases and re-ruptures, tendon retraction, fibrosis, patella alta, and poor tissue quality hinder anatomical restoration [2].

Reconstruction strategies frequently involve tendon autografts combined with augmentation techniques [3]; however, there is no consensus regarding the optimal approach in complex revision scenarios, particularly when associated with bone loss.

We report a case of multiply failed patellar tendon repair with patella alta and significant bone defect of the inferior patellar pole, treated with combined tendon reconstruction and structural bone grafting.

## Case report

A 41-year-old male cattle farmer presented with progressive anterior knee pain. Initial magnetic resonance imaging demonstrated proximal patellar tendinopathy. He subsequently sustained a spontaneous rupture of the patellar tendon, treated with primary repair, which failed within 3 months (Fig. 1).

**Figure 1:**
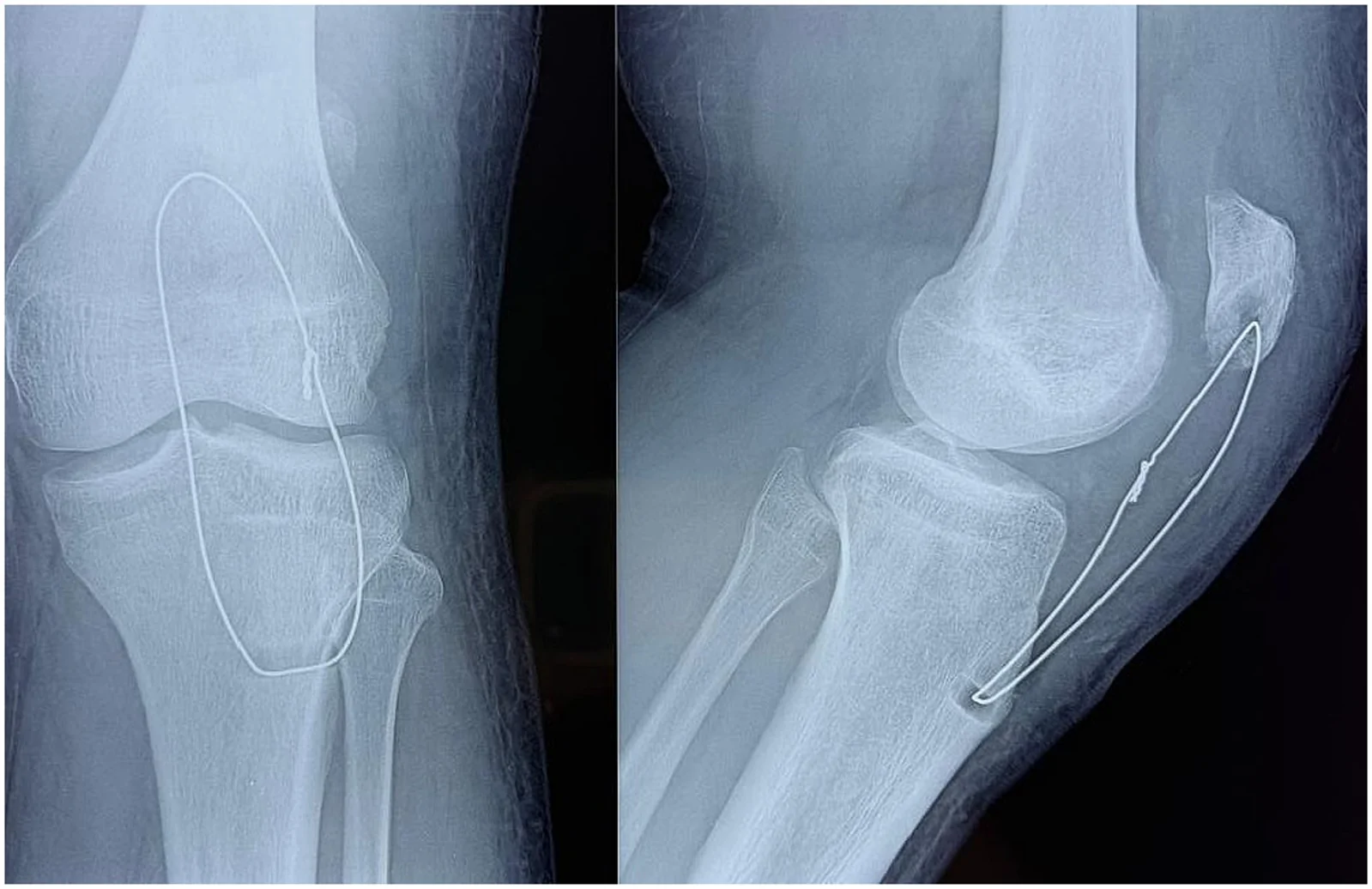
Anteroposterior and lateral radiographs of the left knee showing patellar tendon repair with hamstring tendon augmentation and metallic cerclage extending from the inferior patellar pole to the tibial tuberosity.

Revision surgery using hamstring tendon augmentation and metallic cerclage was performed but evolved to re-rupture, persistent patella alta, and significant osteolysis of the inferior patellar pole and tibial tuberosity (Fig. 2).

**Figure 2:**
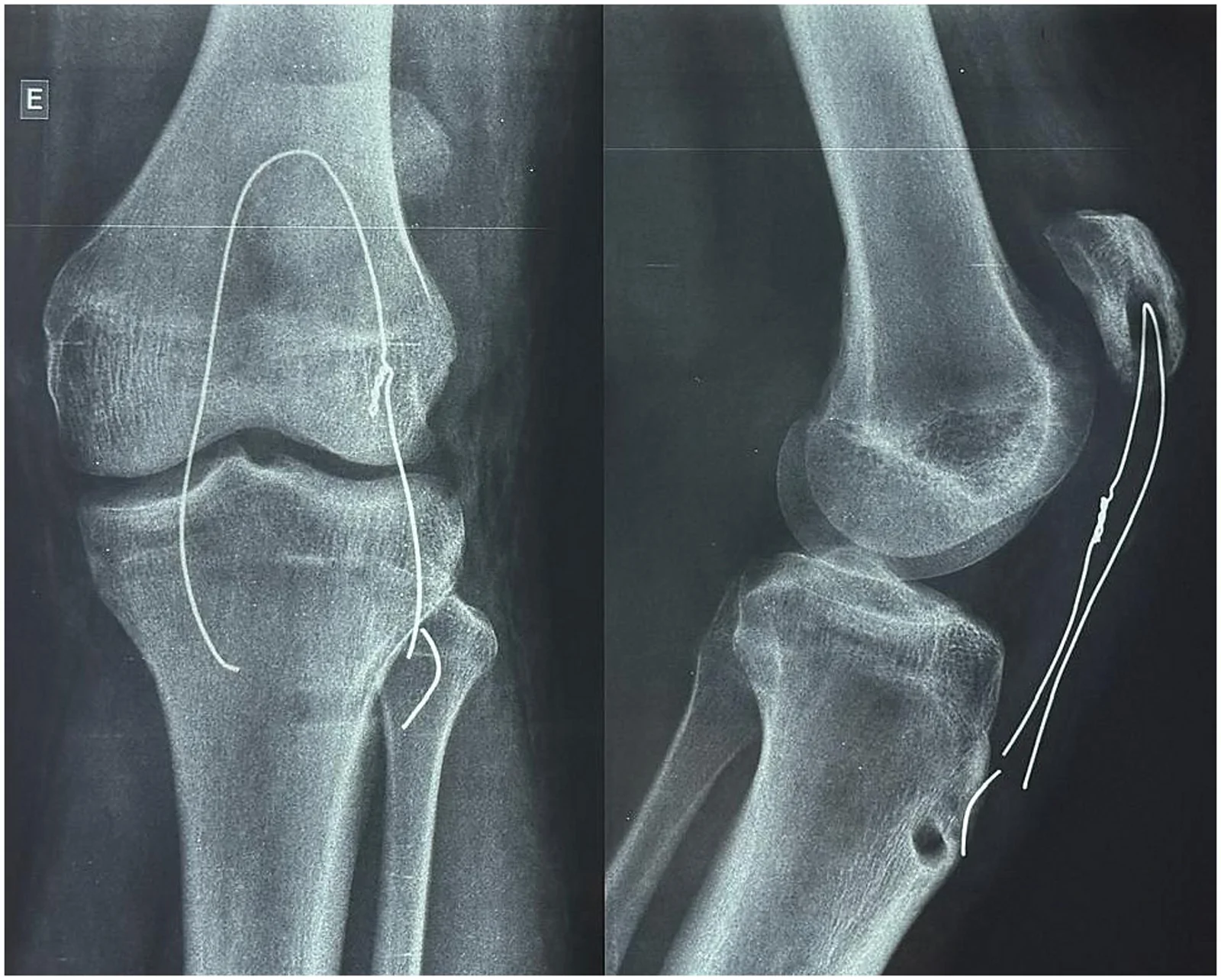
Anteroposterior and lateral knee radiographs showing cerclage wire breakage, persistent patella alta, and osteolysis of the inferior patellar pole and tibial tuberosity.

At presentation, the patient reported pain, instability, and inability to perform daily activities. Functional scores demonstrated severe impairment (IKDC 14%, Lysholm 11). Physical examination showed patella alta (Fig. 3), active extension deficit, reduced range of motion (15°–110°), and quadriceps atrophy. Imaging confirmed tendon insufficiency and bone loss (Figs 3 and 4).

**Figure 3:**
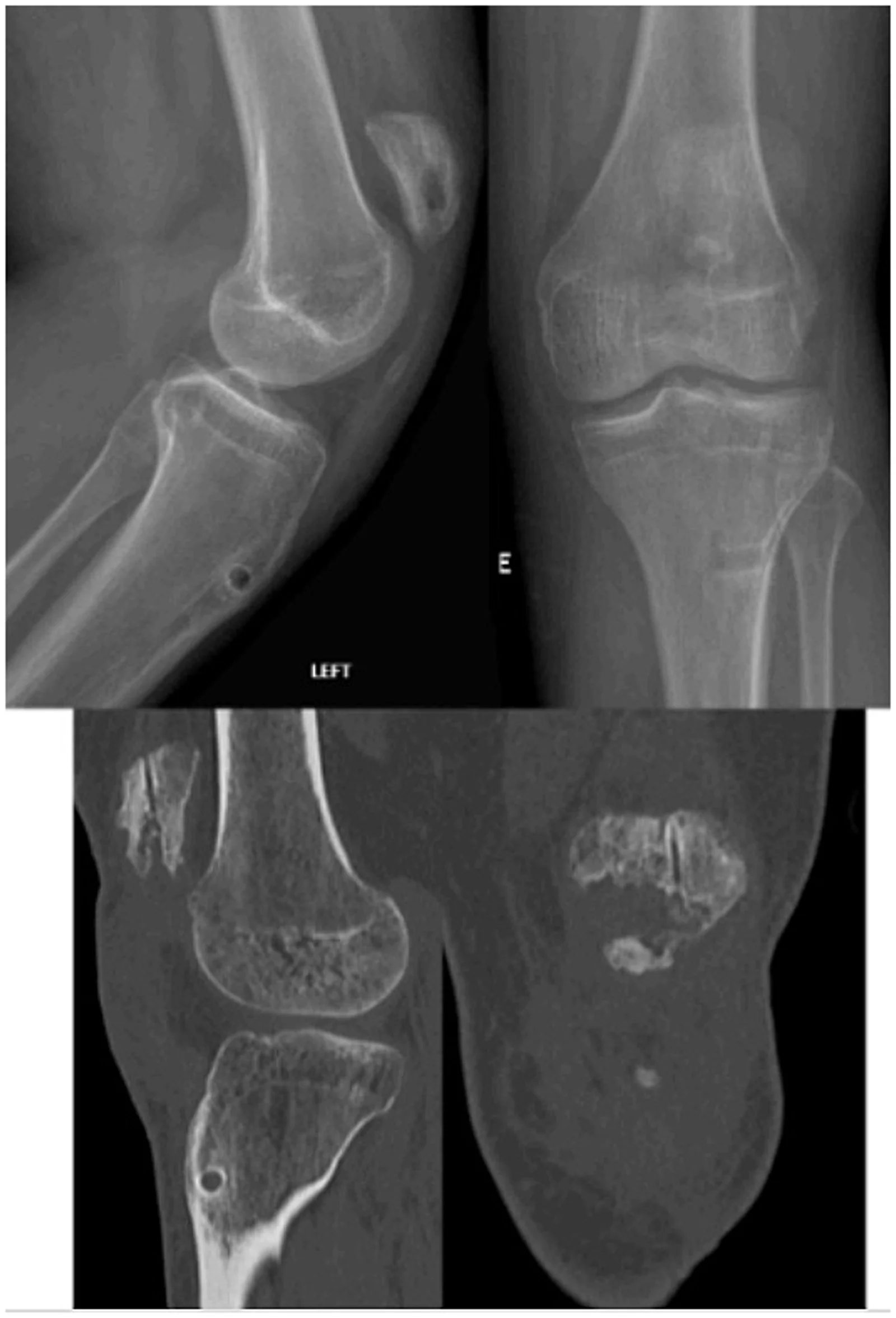
Radiographs demonstrating patella alta, widened previous bone tunnels with osteolysis, and an extensive bony defect of the inferior patellar pole.

**Figure 4:**
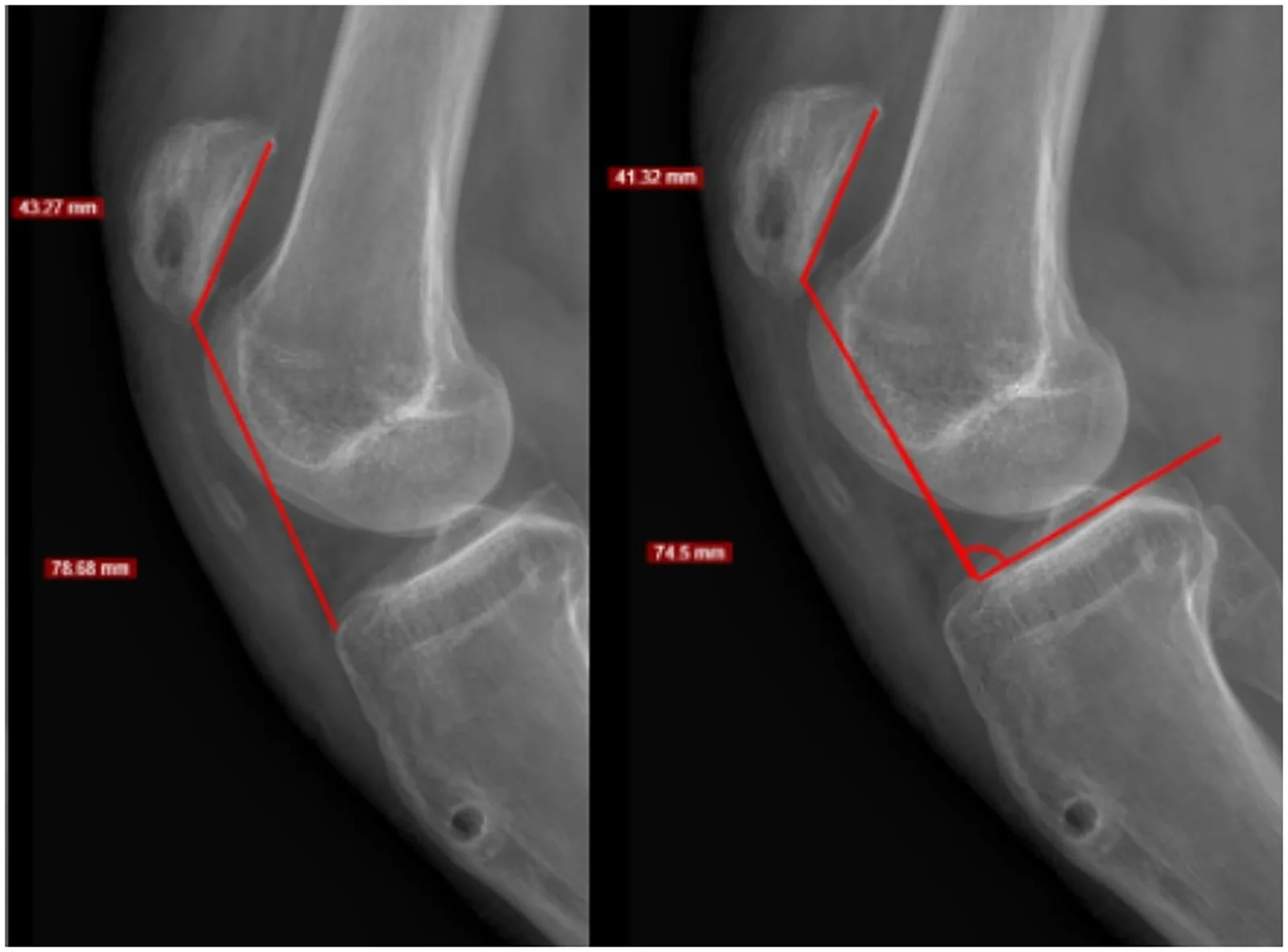
Preoperative patellar height measurements demonstrating a Caton–Deschamps index of 1.81 and Blackburne–Peel index of 1.80.

Reconstruction was performed using suture anchors placed in the patella combined with a structural cortical bone graft harvested from the distal tibia to restore bone support. A peroneus longus tendon autograft was used for augmentation and fixed distally through a new tibial tunnel with an interference screw. The residual tendon was reinforced with Krakow sutures (Fig. 5).

**Figure 5:**
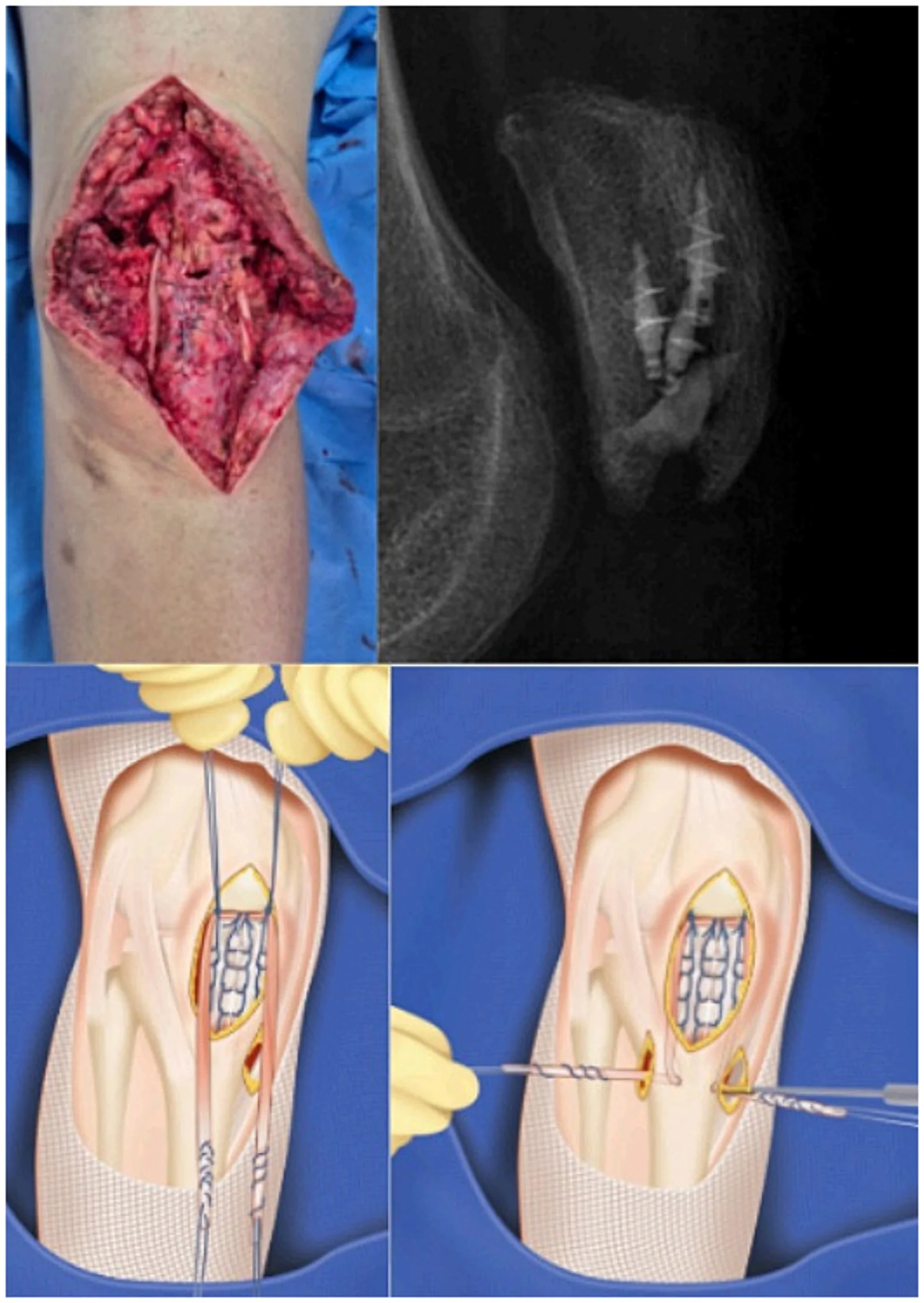
Residual patellar tendon reinforced with an autologous peroneus longus graft and Krakow sutures. Adjacent schematic illustration of the reconstruction technique and postoperative image demonstrating the structural cortical bone graft at the inferior patellar pole.

Postoperative rehabilitation was progressive. At 26 weeks, the patient achieved full active extension, range of motion from 0° to 120°, and substantial functional improvement (IKDC 77%, Lysholm 91, Tegner 5) (Fig. 6). Radiographs demonstrated correction of patellar height (Fig. 7).

**Figure 6:**
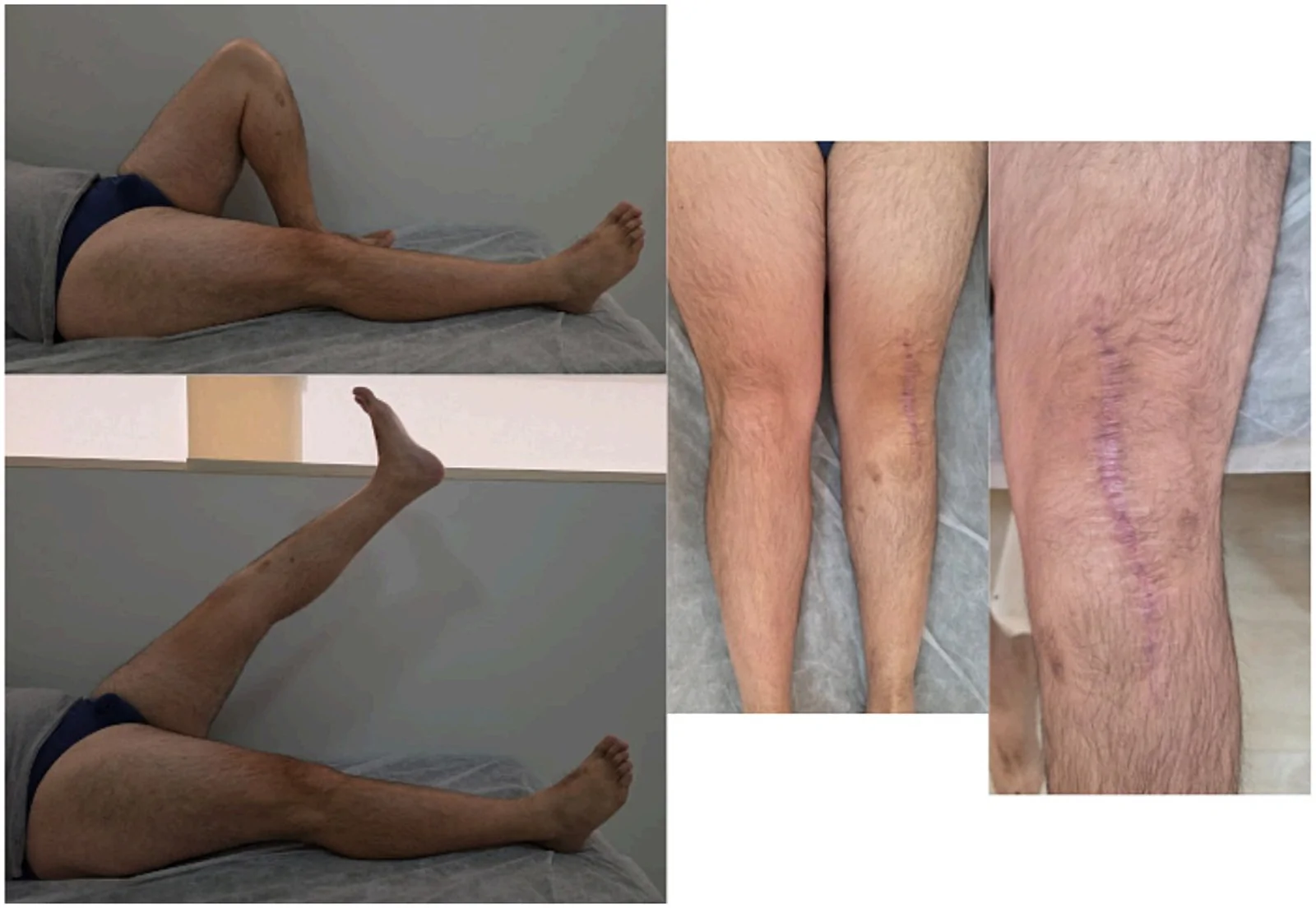
Clinical photographs demonstrating quadriceps muscle trophism and active range of motion of the left knee.

**Figure 7:**
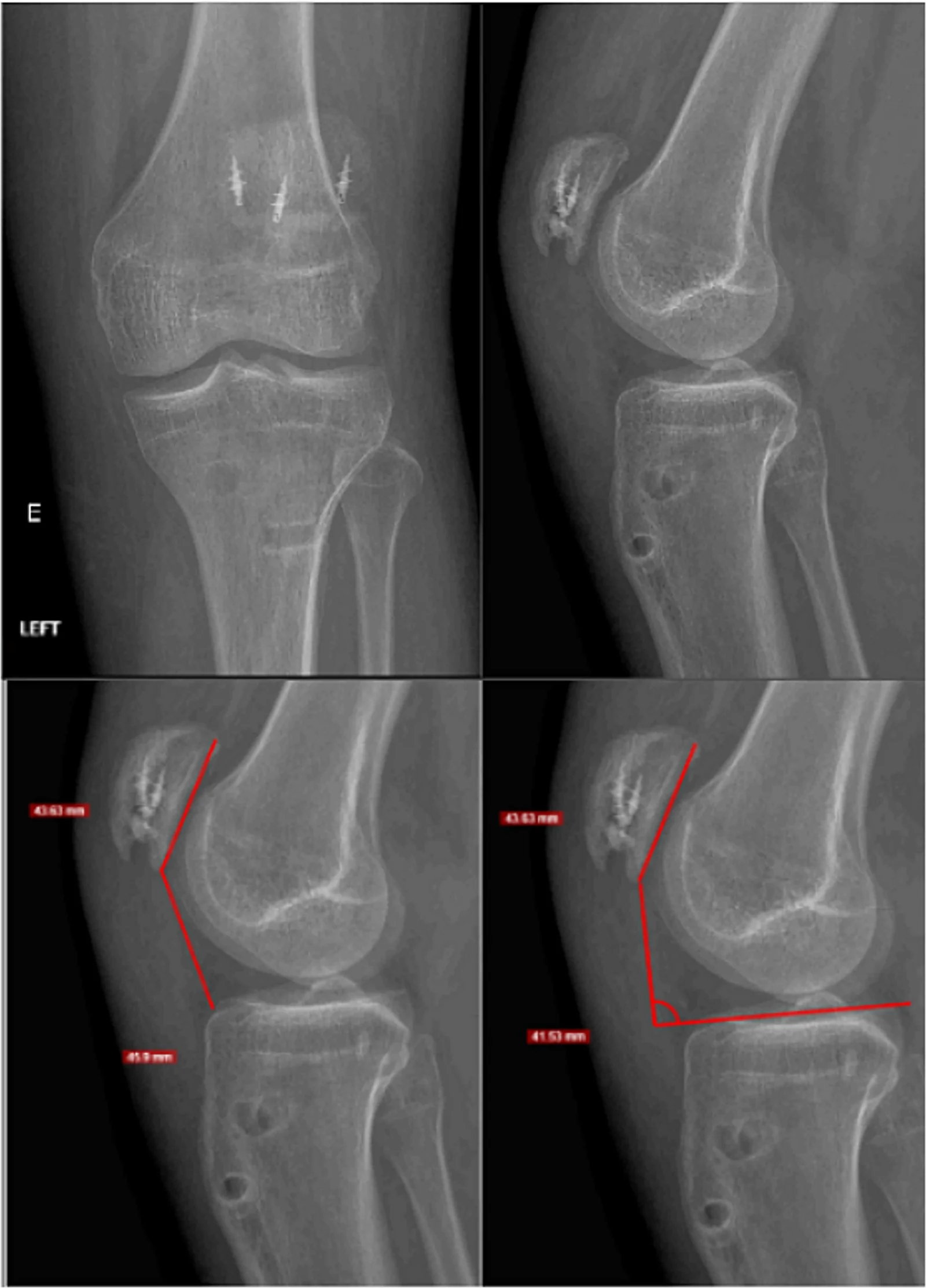
Anteroposterior and lateral radiographs demonstrating postoperative patellar height, with Caton–Deschamps and Blackburne–Peel indices of 1.05 and 0.95, respectively.

## Discussion

This case highlights the challenges of managing complex patellar tendon re-ruptures associated with tendon degeneration, patella alta, and bone loss. In such scenarios, isolated repair is often insufficient, and augmentation strategies become necessary [4, 5].

Autologous grafts remain advantageous due to biological integration [6]. The peroneus longus tendon represents a viable alternative when hamstring tendons are unavailable [7, 8].

A critical aspect in this case was the reconstruction of the inferior patellar pole using a structural cortical bone graft. Bone loss is frequently underestimated but may significantly compromise fixation stability. The graft provided mechanical support, reducing the risk of anchor failure and enabling restoration of patellar height [3, 4].

The favorable short-term outcome suggests that combining tendon augmentation with structural bone reconstruction may improve stability and function in complex revision cases. However, further studies are required to confirm long-term outcomes.
